# Transcriptome-wide analysis of changes in the fetal placenta associated with prenatal arsenic exposure in the New Hampshire Birth Cohort Study

**DOI:** 10.1186/s12940-019-0535-x

**Published:** 2019-11-21

**Authors:** Emily F. Winterbottom, Yuguang Ban, Xiaodian Sun, Anthony J. Capobianco, Carmen J. Marsit, Xi Chen, Lily Wang, Margaret R. Karagas, David J. Robbins

**Affiliations:** 1 Molecular Oncology Program, DeWitt Daughtry Family Department of Surgery, University of Miami Miller School of Medicine, Miami, FL 33136 USA; 2 Sylvester Comprehensive Cancer Center, Miller School of Medicine, University of Miami, Miami, FL 33136 USA; 3 Department of Environmental Health, Rollins School of Public Health at Emory University, Atlanta, GA 30322 USA; 4 Department of Public Health Sciences, Miller School of Medicine, University of Miami, Miami, FL 33136 USA; 5 Department of Human Genetics, Dr. John T. Macdonald Foundation, John P. Hussman Institute for Human Genomics, University of Miami, Miami, FL 33136 USA; 6 Department of Epidemiology, Geisel School of Medicine at Dartmouth, Hanover, NH 03755 USA

**Keywords:** Arsenic, Prenatal, Placenta, RNA-seq, Birth weight, Proteasome

## Abstract

**Background:**

Increasing evidence suggests that prenatal exposure to arsenic, even at common environmental levels, adversely affects child health. These adverse effects include impaired fetal growth, which can carry serious health implications lifelong. However, the mechanisms by which arsenic affects fetal health and development remain unclear.

**Methods:**

We addressed this question using a group of 46 pregnant women selected from the New Hampshire Birth Cohort Study (NHBCS), a US cohort exposed to low-to-moderate arsenic levels in drinking water through the use of unregulated private wells. Prenatal arsenic exposure was assessed using maternal urine samples taken at mid-gestation. Samples of the fetal portion of the placenta were taken from the base of the umbilical cord insertion at the time of delivery, stored in RNAlater and frozen. We used RNA sequencing to analyze changes in global gene expression in the fetal placenta associated with in utero arsenic exposure, adjusting for maternal age. Gene set enrichment analysis and enrichment mapping were then used to identify biological processes represented by the differentially expressed genes. Since our previous analyses have identified considerable sex differences in placental gene expression associated with arsenic exposure, we analyzed male and female samples separately.

**Results:**

At FDR < 0.05, no genes were differentially expressed in female placenta, while 606 genes were differentially expressed in males. Genes showing the most significant associations with arsenic exposure in females were *LEMD1* and *UPK3B* (fold changes 2.51 and 2.48), and in males, *FIBIN* and *RANBP3L* (fold changes 0.14 and 0.15). In gene set enrichment analyses, at FDR < 0.05, a total of 211 gene sets were enriched with differentially expressed genes in female placenta, and 154 in male placenta. In female but not male placenta, 103 of these gene sets were also associated with reduced birth weight.

**Conclusions:**

Our results reveal multiple biological functions in the fetal placenta that are potentially affected by increased arsenic exposure, a subset of which is sex-dependent. Further, our data suggest that in female infants, the mechanisms underlying the arsenic-induced reduction of birth weight may involve activation of stress response pathways.

## Background

An abundance of epidemiological studies have linked prenatal arsenic exposure to a range of adverse infant health outcomes, including spontaneous abortion and neonatal mortality; reduced birth weight; and increased risk of infections in infanthood (reviewed in [1]). Although the effects of high-level exposure are more severe, reports suggest that even levels close to the World Health Organization’s recommended drinking water limit of 10 μg/L, which are estimated to affect millions of people worldwide [2], can be harmful. The New Hampshire Birth Cohort Study (NHBCS) is an ongoing cohort study that was initiated in 2009 to elucidate the effects of such common levels of prenatal exposure to arsenic on maternal and infant health [3]. Participants in the study are pregnant women who use an unregulated private water supply in a US region with low to moderate groundwater arsenic levels. Analyses of the NHBCS have already revealed associations of arsenic exposure with fetal growth measures including birth weight [4]; childhood infections [5]; and various physiological and molecular changes in both the cord blood and placenta [6–10].

Birth weight, a measure of fetal growth, can be an important indicator of risk of both childhood and adult disease conditions including neurocognitive disorders, diabetes, hypertension, and renal disease [11]. Several epidemiological studies have found decreased birth weight associated with greater in utero exposure to arsenic [12, 13], and this has been observed even at relatively low levels of exposure [4, 14]. However, thus far, the mechanism by which arsenic impairs fetal growth is unclear. We have addressed this question by analyzing how arsenic exposure is associated with gene expression in the fetal placenta. The fetal placenta plays a central role in the regulation of fetal growth, controlling the flow of nutrients and oxygen, producing essential hormones, and acting as a protective barrier. Moreover, it accumulates arsenic to up to three times the level in maternal blood [15]. Thus, it is likely that the effects of arsenic on the fetal placenta play a major role in the etiology of low birth weight caused by prenatal exposure. We used RNA sequencing (RNA-seq) as an unbiased, transcriptome-wide approach to identify genes whose expression in the fetal placenta is related to arsenic exposure. Further, using bioinformatic analyses, we identified biological processes related to arsenic exposure, and to birth weight, and used these data to identify potential mechanisms through which arsenic impacts fetal placental function to reduce fetal growth and birth weight. In this initial study, we focused on a group of 46 infants with the lowest and highest levels of prenatal arsenic exposure among a subcohort of the NHBCS. Based on the sexual dimorphism observed in previous analyses of the NHBCS [16, 17], we analyzed the placentas of male and female infants separately.

## Materials and methods

### The New Hampshire birth cohort study (NHBCS)

The study cohort was selected from a subcohort of 312 mother-child pairs who were enrolled in the New Hampshire Birth Cohort Study (NHBCS) [3] between February 2012 and September 2013. Participants were English-speaking, mentally competent women between 18 and 45 years of age, pregnant with a singleton infant, whose home water supply was from a private, unregulated well at their home. Demographic data, pregnancy history and outcome, and lifestyle factor information were collected using prenatal and delivery records and questionnaires. All subjects provided written informed consent in accordance with the requirements of the Institutional Review Board of Dartmouth College.

### Study cohort

For this study, we initially aimed to select 12 infants of each sex with the highest prenatal arsenic exposure within our NHBCS subcohort, and the 12 infants of each sex with the lowest exposure, based on maternal urinary arsenic levels excluding arsenobetaine (U-As, as detailed under “Urine sample collection and arsenic measurement” below). Power analysis, performed using the RNASeqPower R package [18], indicated adequate statistical power (80%) to detect a 2.1-fold change in gene expression using this sample size. One male sample was later found to not have an available urine sample, and one female sample (with low arsenic exposure) was found to be an outlier by principal component analysis of gene expression, as described below. After this exclusion, median average maternal gestational U-As levels were as follows: male high exposure group: 21.04 μg/L (IQR 25.55 μg/L); male low exposure group: 0.67 μg/L (IQR 0.31 μg/L); female high exposure group: 13.81 μg/L (IQR 8.87 μg/L); female low exposure group: 0.74 μg/L (IQR 0.21 μg/L). Other demographic details are provided in Table 1.

**Table 1:** Demographic details of the study cohort

	Males		Females		NHBCS
	High exposure	Low exposure	High exposure	Low exposure	
Number of pregnant women	11	12	12	11	312
Gestational age (wks)	39.76 (1.1)	39.88 (1.0)	38.95 (1.5)	39.41 (1.6)	39.4 (1.5)
Maternal age at enrollment (yrs)	31.73 (4.3)	31.25 (4.5)	30.23 (5.5)	30.72 (3.0)	31.8 (4.8)
Number ever smoked during pregnancy	0 (0)^a^	0 (0)^a^	0 (0)^a^	3 (27.3)^a^	18 (5.8)^a^
Infant birth weight (kg)	3.43 (0.6)	3.49 (0.4)	3.21 (0.6)	3.19 (0.4)	3.4 (0.5)
Total urinary arsenic (U-As, μg/L)	21.04 (25.6)^b^	0.67 (0.3)^b^	13.81 (8.9)^b^	0.74 (0.2)^b^	3.7 (4.1)^b^

Values are presented as means (SD), ^a^number (%), or ^b^median (interquartile range)

### Arsenic exposure assessment

For assessment of prenatal arsenic exposure in this study, we used total maternal urinary arsenic, excluding arsenobetaine (U-As), measured from spot urine samples at mid-gestation. An advantage of using this measure of exposure is that, due to the relatively high levels of arsenic in urine, we were able to measure species levels individually and remove arsenobetaine, which is considered non-toxic [19]. To minimize variation in arsenic intake from household water during gestation, participants were selected for the NHBCS who had not changed residence since their last menstrual period, and were not planning to move residence before delivery. Household water arsenic was previously shown to be strongly associated with maternal urinary arsenic in the NHBCS [3], consistent with it being a major source of exposure.

### Urine sample collection and arsenic measurement

Details of sample collection and arsenic measurement have been described previously [6]. Briefly, maternal spot urine samples were collected at approximately 24–28 weeks of gestation, into acid-washed containers containing diammonium diethyldithiocarbamate to stabilize trivalent methyl arsenic species [20], and frozen at − 80 °C until analysis (within 24 h of collection). Samples were analyzed for levels of five individual arsenic species: arsenite (As^III^), arsenate (As^V^), dimethylarsinic acid (DMA^V^), monomethylarsonic acid (MMA^V^), and arsenobetaine, using high-performance liquid chromatography inductively coupled plasma mass spectrometry (ICP-MS). The detection limits were: 0.15 μg/L for As^III^, 0.10 μg/L for As^V^, 0.14 μg/L for MMA, and 0.11 μg/L for DMA. Numbers of samples below the detection limits were as follows: As^III^: 29, As^V^: 0, DMA^V^: 30, and MMA^V^: 22. We calculated the sum of As^III^, As^V^, DMA^V^ and MMA^V^, with arsenobetaine excluded, as it is considered non-toxic and is not metabolized, and used the resulting value, denoted U-As, as a measure of arsenic exposure. Proportions of different arsenic species did not vary significantly between high and low groups, except in the case of As^V^, which was slightly higher among low exposure than high exposure males (*P* = 0.032, see Additional file 1).

### Placenta biopsy and gene profiling

Placental biopsies were taken by medical staff in the delivery room. Biopsies measured approximately 1 cm deep and 1–2 cm across, and were taken at the base of the umbilical cord insertion to minimize heterogeneity. Any maternal decidua was removed. Samples were immediately placed in tubes containing RNAlater (Life Technologies), and then frozen at − 80 **°**C within 24 h. Total RNA was extracted using the RNA/DNA extraction kit (Norgen Biotek, Thorold, ON), quantified using a NanoDrop spectrophotometer, and stored at − 80 °C. RNA quality was determined using an Agilent 2100 Bioanalyzer, and global gene expression analysis was performed by RNA sequencing (RNA-seq) at the Oncogenomics Core Facility at the University of Miami. Specifically, TruSeq Stranded Total RNA-seq Library Prep kits (Illumina) were used to convert total RNA to cDNA libraries, which were then sequenced using the Genome Analyzer IIx system (Illumina). The male and female RNA-seq assays were performed separately; however, identical procedures were used for both assays.

### Bioinformatic analysis

**Fig. 1:**
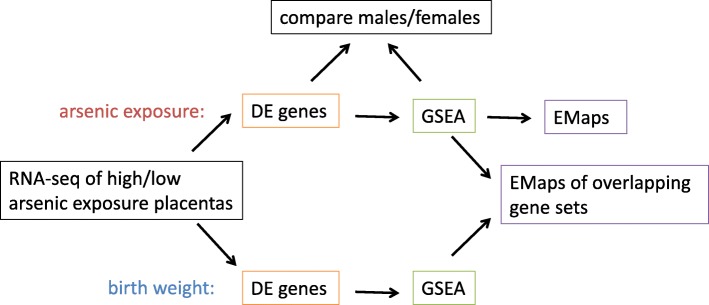
RNA-seq data analysis. Schematic diagram summarizing the analyses described in this report. DE; differentially expressed, GSEA; gene set enrichment analysis, EMap; enrichment map

A summary of the analysis pathway is provided in Fig. 1. Specifically, sequencing fragments were aligned to the human reference genome (GRCh38) using the STAR algorithm [21], and reads mapping to genomic features (transcribed RNA) were counted using featureCounts [22]. Differential expression was assessed using DESeq2 [23]. Specifically, samples were dichotomized into high and low arsenic exposure groups. A linear model: raw read counts ~ groups + maternal age at enrollment + batch was then fitted to each gene, and Wald significance tests and their corresponding *p*-values were used to assess differences in expression related to arsenic exposure, adjusted for maternal age at enrollment and assay batch [24]. For analyses of differences in expression related to birth weight, birth weight (in kilograms) was considered as a continuous variable. Gestational age, enrollment age and assay batch were also included as covariates in the linear models for association tests with birth weight. The adjustments (excluding assay batch) were based on previous analyses of the NHBCS, in which a range of variables (maternal age, maternal smoking status (never, former, current), maternal education level, infant birth weight, infant sex and gestational age) were assessed as potential confounders using a series of linear regression models [25]. In our data, we found the above variables to be associated with U-As/birth weight and gene expression. To account for multiple comparisons, we computed the False Discovery Rate (FDR) [26].

To further evaluate the potential molecular and functional effects of arsenic exposure, we performed gene set enrichment analysis (GSEA) [27]. This involved identifying gene sets (representing biological processes) enriched with differentially expressed genes. The “canonical pathways” (CP) gene set collection from the Molecular Signatures Database (MSigDB), comprising 1330 gene sets, was used. Gene sets were ranked by normalized enrichment score (NES), a linear measurement of the degree to which a gene set is overrepresented at the top or bottom of a list of genes ranked according to their differential expression [27]. Enrichment Maps [28] were then generated to visualize the major biological themes of the GSEA results, by grouping the identified gene sets into clusters based on common genes.

RNA-seq quality control data are provided in Additional file 2. An average of 58.2 million and 68.0 million reads were sequenced from female and male placenta samples respectively. Of these, 96.55 and 98.06% respectively were aligned to the reference genome, and 49.21 and 46.09% respectively were assigned to genes (mapped to transcribed RNA). Principal component analysis (PCA) of the gene expression data revealed one female sample (with low arsenic exposure) to be an outlier (Additional file 3A); this sample was therefore excluded from subsequent analyses. PCA did not reveal any outliers among the male samples (Additional file 3B), but showed that the male and female samples clustered separately (Additional file 3C).

## Results

The RNA-seq data were analyzed as summarized in Fig. 1. First, we identified genes that were differentially expressed (DE) between high- and low-U-As groups (Additional file 4). At *P* < 0.05, 1748 genes were DE in female placenta, and 2438 genes in male placenta. The top DE genes were *LEMDI* and *UPK3B* in females (fold changes 2.51 and 2.48), and *FIBIN* and *RANBP3L* in males (fold changes 0.14 and 0.15). A total of 458 DE genes at *P* < 0.05 were common to males and females (data not shown). After FDR adjustment for multiple comparisons, no genes were DE in females at FDR < 0.05, while 606 genes were differentially expressed in males (Additional file 4). To validate these results, we performed qPCR for four of the top DE genes in females using new tissue samples from the same placentas, and found that the results were largely in keeping with our RNA-seq data (Additional file 5).

Next, we performed gene set enrichment analysis (GSEA) to identify gene sets enriched with DE genes in our high versus low U-As groups. Such gene sets represent biological processes associated with arsenic exposure. At a significance level of FDR < 0.05, in females, 87 gene sets were upregulated, and 124 gene sets were downregulated, while in males, 7 gene sets were upregulated, and 146 gene sets were downregulated (Additional file 6A-D). Table 2 shows the top five gene sets in each group, based on normalized enrichment scores (NES). In females (Table 2A), the top upregulated gene sets were related to cellular respiration and the ubiquitin proteasome system, while downregulated gene sets involved protein synthesis and the extracellular matrix. In males (Table 2B), the top upregulated gene sets were related to the unfolded protein response and endocytosis/recycling of cell surface proteins, while downregulated gene sets involved the extracellular matrix, as in females, and smooth muscle contraction.

**Table 2:** Top gene sets associated with arsenic exposure

Gene set	NES
A. Female	
Upregulated	
KEGG OXIDATIVE PHOSPHORYLATION	3.86
REACTOME RESPIRATORY ELECTRON TRANSPORT ATP SYNTHESIS BY CHEMIOSMOTIC COUPLING AND HEAT PRODUCTION BY UNCOUPLING PROTEINS	3.76
REACTOME CLASS I MHC MEDIATED ANTIGEN PROCESSING PRESENTATION	3.75
REACTOME ANTIGEN PROCESSING UBIQUITINATION PROTEASOME DEGRADATION	3.71
REACTOME MEMBRANE TRAFFICKING	3.70
Downregulated	
NABA CORE MATRISOME	−4.59
REACTOME PEPTIDE CHAIN ELONGATION	−4.57
KEGG RIBOSOME	−4.40
REACTOME INFLUENZA VIRAL RNA TRANSCRIPTION AND REPLICATION	−4.12
REACTOME SIGNALING BY GPCR	−3.87
B. Male	
Upregulated	
REACTOME UNFOLDED PROTEIN RESPONSE	2.17
REACTOME INSULIN RECEPTOR RECYCLING	2.10
REACTOME ACTIVATION OF CHAPERONE GENES BY XBP1S	2.04
REACTOME TRANSFERRIN ENDOCYTOSIS AND RECYCLING	2.01
REACTOME ENDOSOMAL SORTING COMPLEX REQUIRED FOR TRANSPORT ESCRT	1.99
Downregulated	
NABA CORE MATRISOME	−2.78
NABA ECM GLYCOPROTEINS	−2.54
PID INTEGRIN1 PATHWAY	− 2.37
KEGG VASCULAR SMOOTH MUSCLE CONTRACTION	−2.36
NABA COLLAGENS	−2.36

*NES* normalized enrichment score

We then compared the male and female GSEA results (Additional file 6E). Six gene sets were upregulated and 61 gene sets were downregulated at high U-As in both male and female placentas.

To better characterize these results, we generated enrichment maps (EMaps) [28]. These maps group the gene sets identified by GSEA into clusters, to identify the major biological themes associated with arsenic exposure in female and/or male fetal placentas (Additional files 7 and 8). Comparison of the clusters in each EMap revealed both similarities and differences between the sexes. For example, in both male and female placentas, gene set clusters related to the extracellular matrix (ECM) and muscle contraction were downregulated, and a cluster related to the unfolded protein response was upregulated. In contrast, several gene set clusters were unique to females, in particular a large upregulated cluster related to proteasomal degradation, while male placentas showed downregulation of a large gene set cluster related to a range of cell processes/components including G protein coupled receptor (GPCR) signaling, and T cell receptor activation, only a subset of which was also downregulated in females. In addition, clusters related to autoimmune responses/inflammation, and interferon gamma signaling, were largely unique to male placentas. These data suggest that multiple cellular functions in the fetal placenta may be altered by increased arsenic exposure at the common environmental levels encountered in our study, and that a subset of these potential effects are fetal sex-dependent.

**Fig. 2:**
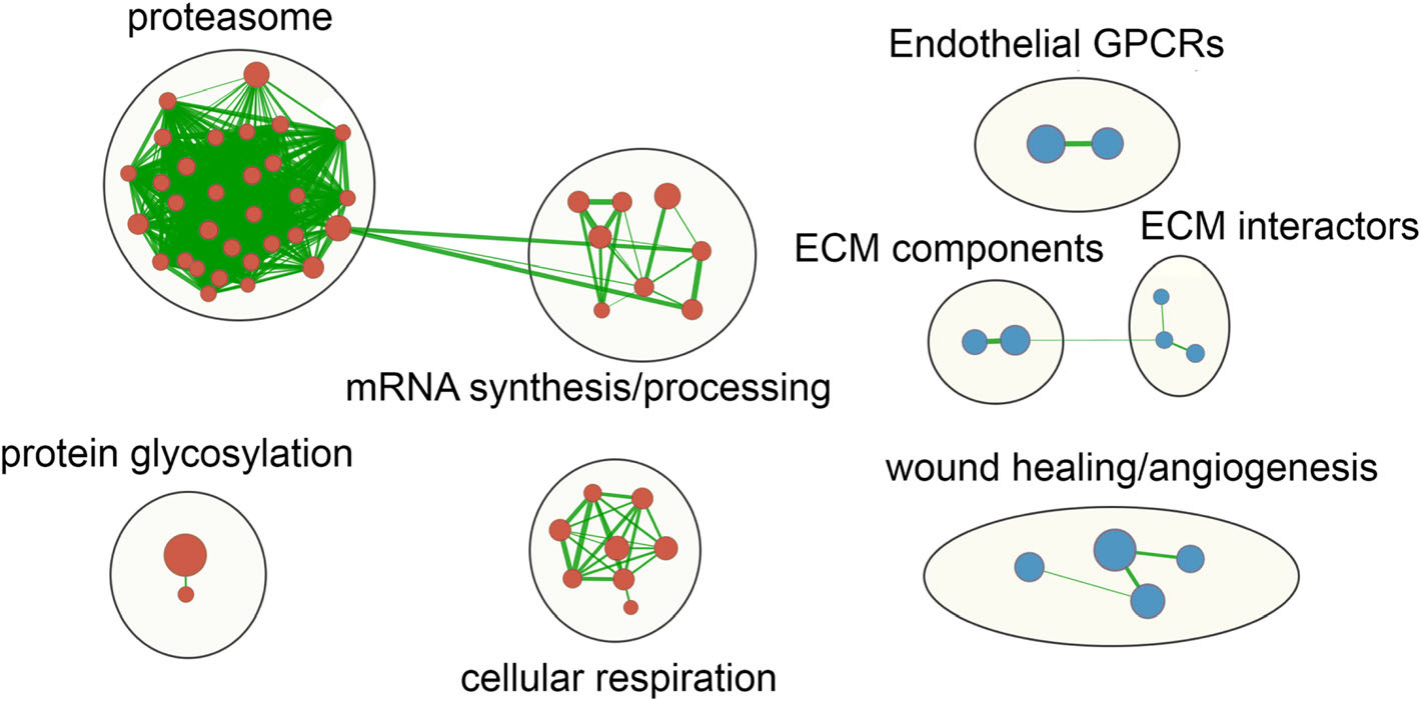
Gene sets differentially related to arsenic exposure and birth weight in female fetal placenta. Enrichment map of gene sets that are associated with increased arsenic exposure and reduced birth weight in female placenta (with a significance level of FDR < 0.05). Clusters thus represent biological themes that may underlie female fetal growth inhibition by arsenic. Red circles; upregulated gene sets, blue circles; downregulated gene sets, large yellow circles; gene set clusters, green lines indicate overlapping genes between gene sets. Clusters were labeled based on the most common themes of the gene sets they comprise. Singleton gene sets are omitted (see Additional file 10A for a complete list of gene sets)

Previous studies have indicated that higher prenatal arsenic exposure may decrease birth weight [4, 12–14]; however, the underlying mechanisms are unknown. Thus, we next used our RNA-seq data to identify biological processes that may mediate adverse effects of arsenic on fetal growth, by comparing the gene sets identified by GSEA at high versus low U-As with those that were associated with birth weight (as a continuous variable). In female placenta, at FDR < 0.05, 183 gene sets were associated with birth weight (Additional file 9A), and 103 of these were also associated with U-As (Additional file 10A). Of these, 51 gene sets were upregulated and 16 downregulated at high U-As and low birth weight, representing a total of 66 biological processes potentially mediating female birth weight - arsenic exposure associations. The top 10 of these included downregulation of the core matrisome (ECM components), and upregulation of electron transport chain and proteasome components (Table 3). An EMap was generated of the 66 gene sets, revealing 7 gene-set clusters (Fig. 2). The upregulated clusters were related to the ubiquitin proteasome pathway, cellular respiration, mRNA synthesis/processing, and protein glycosylation; while the downregulated clusters involved ECM components and interactors, endothelial G-protein-coupled receptors, and growth factors involved in wound healing and angiogenesis.

**Table 3:** Top 10 gene sets enriched at high arsenic exposure and reduced birth weight in female fetal placenta

Gene set	U-As NES	Birth weight NES	U-As FDR value	Birth weight FDR value
NABA CORE MATRISOME	−4.59	3.49	0	0
KEGG OXIDATIVE PHOSPHORYLATION	3.86	−3.45	0	6.51 × 10^−7^
REACTOME RESPIRATORY ELECTRON TRANSPORT ATP SYNTHESIS BY CHEMIOSMOTIC COUPLING AND HEAT PRODUCTION BY UNCOUPLING PROTEINS	3.76	− 3.19	0	2.87 × 10^−6^
REACTOME TCA CYCLE AND RESPIRATORY ELECTRON TRANSPORT	3.69	− 3.32	0	2.17 × 10^− 6^
REACTOME HIV INFECTION	3.25	−3.61	1.22 × 10^−5^	0
REACTOME VIF MEDIATED DEGRADATION OF APOBEC3G	3.1	−3.4	2.93 × 10^− 5^	1.20 × 10^− 6^
REACTOME REGULATION OF ORNITHINE DECARBOXYLASE ODC	3.1	−3.19	3.13 × 10^− 5^	2.51 × 10^− 6^
KEGG PROTEASOME	3.09	−3.14	3.11 × 10^− 5^	3.86 × 10^− 6^
REACTOME SCF BETA TRCP MEDIATED DEGRADATION OF EMI1	3.08	−3.23	3.10 × 10^− 5^	2.24 × 10^− 6^
KEGG HUNTINGTONS DISEASE	3.01	−3.15	4.53 × 10^− 5^	3.94 × 10^− 6^

*NES* normalized enrichment score

In male fetal placenta, 147 gene sets were associated with birth weight at FDR < 0.05 (Additional file 9B), and 100 of these were also negatively associated with U-As (Additional file 10B). However, no gene sets were positively associated with U-As, and none showed differential associations with U-As and birth weight. Therefore, in contrast to females, this analysis did not reveal any biological processes that might mediate decreases in birth weight related to arsenic exposure in male fetal placenta.

To explore the sexual dimorphism we observed in the biological processes linking arsenic exposure and birth weight, we compared the gene sets associated with birth weight in male and female placentas (Additional file 9C). Interestingly, at FDR < 0.05, 22 gene sets were associated with birth weight in both sexes, but 16 of these showed opposite directions of association, namely negative in males but positive in females. Among these 16 gene sets, 14 were also negatively associated with U-As in both sexes, and comprised all of the downregulated gene-set clusters potentially linking increased arsenic exposure with lower female infant birth weight shown in the EMap in Fig. 2. These clusters were related to ECM components and interactors, endothelial G-protein-coupled receptors, and wound healing and angiogenesis.

## Discussion

Our RNA-seq analysis identified numerous genes whose expression in fetal placenta appeared to associate with arsenic exposure in a sex-dependent manner, although these associations did not withstand adjustment for multiple comparisons in females. Further, GSEA and enrichment mapping revealed both common and sex-specific biological processes, represented by gene sets and gene-set clusters, that were associated with arsenic exposure. The most significant processes common to both sexes included upregulation of the unfolded protein response (UPR). The UPR is activated by endoplasmic reticulum (ER) stress, an accumulation of unfolded proteins in the ER. Arsenic has been shown in human and mouse cell culture studies to activate the UPR [29, 30], likely by interfering with oxidative protein folding [31]. Also common to both sexes was downregulation of ECM components, endothelial GPCRs, and wound healing and angiogenesis. These results are somewhat reminiscent of a study in mice that found reduced expression of ECM genes, and disruption of arterial ECM, in the heart and lungs following chronic arsenic exposure [32]. These gene sets were also inversely associated with birth weight in female placentas, suggesting that reduced expression of ECM components, endothelial GPCRs, and factors involved in wound healing and angiogenesis may contribute to lower female infant birth weight caused by arsenic exposure. However, interestingly, we found that, in male placentas, these gene sets were positively associated with birth weight. One explanation for this is that these responses are not alone sufficient to affect fetal growth, but that, in the females in our analysis, they have occurred in parallel with other functional changes that directly reduce fetal growth.

Such functional changes may be represented by four gene set clusters that were upregulated with higher arsenic exposure and lower birth weight in female, but not male, placentas. These clusters represent the proteasome, cellular respiration, mRNA synthesis/processing, and protein glycosylation. Some of these pathways may be activated in response to arsenic-induced oxidative stress. Numerous studies have found arsenic to induce oxidative stress and reactive oxygen species production (reviewed in [33, 34]). To counteract the effects of oxidative stress, cells activate the antioxidant response pathway. This involves activation of the NFE2L2 (Nrf2) transcription factor, leading to transcription of a suite of genes that have antioxidant response elements (AREs) in their promoters. Notably, these include many subunits of the 26S proteasome, which is upregulated to remove oxidatively damaged proteins [35]. NFE2L2 has also been shown to stimulate mitochondrial biogenesis [36], which may explain the increased expression of electron transport chain components. Increased transcription and synthesis of NFE2L2 targets may also explain the observed upregulation of mRNA synthesis and processing, and protein glycosylation pathways. Increased protein glycosylation may also be a feedback response to impaired protein folding in the ER in response to arsenic, as mentioned above.

Our analyses also identified some biological processes that were associated with arsenic exposure exclusively in male placentas. Among the most significant of these were upregulation of genes related to transferrin endocytosis, and downregulation of those related to autoimmune responses/inflammation, and interferon gamma signaling. Transferrin is an iron transport protein that is expressed in both the cytotrophoblast and syncytiotrophoblast of the fetal placenta [37–39]. In support of our findings, a previous study reported increased transferrin expression in the syncytiotrophoblast of pregnancies complicated by maternal drug abuse, gestational diabetes or pregnancy-induced hypertension, suggesting that this may represent a response to intrauterine stress [37]. Placental expression of the transferrin receptor was also increased in conditions of iron deficiency [40]. Interferon gamma (IFNG) is a proinflammatory cytokine that is produced by various immune cells, including natural killer and CD4+ T helper 1 (Th1) cells [41], and plays an central role in the development of autoimmunity [42]. IFNG and its receptors are also expressed by the trophoblast of the fetal placenta; however this tissue shows a dampened response to IFNG [41], and the fetal/neonatal immune system tends to be tolerogenic and Th2-biased, with minimal IFNG production, to avoid responses to maternal alloantigens [43, 44]. Our results appear to suggest that, in male fetal placenta, arsenic exposure increases this anti-inflammatory bias. This finding aligns with a previous study of the NHBCS, which found increased numbers of Th2-type cells in cord blood at high arsenic exposure, although sex differences were not observed [10]. Studies of adults chronically exposed to arsenic have also shown immune effects, including reduced expression of IFNG [45]. Arsenic exposure, including prenatal exposure [5, 46], has been associated with increased susceptibility to various infections, and some studies have recorded sex differences in such associations, with males tending to be more susceptible (reviewed in [47]).

Limitations of this study include the small sample size, the large range of U-As values within the “high” and “low”-arsenic groups, and the variation in these ranges between male and female placenta samples. In addition, RNA sequencing of the male and female samples were performed separately. Therefore, although the samples were treated and analyzed in an identical manner, it may be that a subset of the differences observed is due to experimental variation, rather than sex. A further limitation is that, in this study, we did not examine levels of other metal toxicants in maternal urine, so cannot rule out the possibility of a confounding effect by other elements. Another potential confounding factor is that the participants are exposed to arsenic from different sources, e.g. rice, seafood, and drinking water, which contains different proportions of arsenic species and therefore may have different effects. The relative proportion of As^V^ in males was slightly higher in the low arsenic group (Additional file 1), which may have influenced some of the noted associations. Models were adjusted for RNA-seq batch, maternal age, and in the birth weight analysis, gestational age. Additional potential confounders, i.e., maternal smoking status, and maternal education level were not found to significantly associate with U-As in our previous analyses of the NHBCS [25]. Another important point to acknowledge is that we are using gene expression after delivery to draw conclusions about prenatal gene expression. There are likely to be numerous changes occurring in the fetal placenta within this period, and therefore caution must be taken in the interpretation of our findings.

In this study, we chose to use maternal urinary arsenic (excluding arsenobetaine), measured at mid-gestation, as our measure of prenatal arsenic exposure. To minimize variation in arsenic exposure between mid-gestation and delivery, participants were selected who did not plan to move residence between mid-gestation and delivery. Thus, the household water supply, a major component of overall arsenic exposure [3], remained constant. An advantage of using urinary arsenic as the exposure measure was the ability to measure individual species, and thus remove arsenobetaine from our calculation, which is considered non-toxic [19]. In contrast, placental arsenic is present in much lower levels, and therefore, when it is used for exposure assessment, arsenobetaine cannot be accounted for. However, clearly, mid-gestational U-As has a number of disadvantages: primarily the time difference between urine sampling and placental expression analysis, and the fact that only a single sample was taken, as well as the increased metabolism of arsenic during pregnancy [48]. These factors must be acknowledged as important limitations of the current study. Future studies should include similar studies using placental arsenic for assessment of prenatal exposure.

## Conclusions

Our results suggest that common levels of arsenic exposure are associated with multiple changes in the human placental transcriptome, a subset of which was sex-specific. Further, we identified some potential sex-dependent mechanisms for the known adverse effects of arsenic on birth weight. Overall, our findings offer insights into potential mechanisms through which prenatal arsenic exposure may impact the fetal placenta in a sex-dependent manner to affect fetal health and development, which may be further explored in future studies.

## Supplementary information

**Additional file 1. **Relative proportions of arsenic species in high and low arsenic groups. Upper and lower ends of boxes indicate the 25th and 75th percentiles, respectively, and black band represents the median. Error bars represent minimum and maximum values, excluding outliers, which are depicted as dots. *P* values are based on a Wilcoxon signed rank test.

**Additional file 2.** RNA-seq quality control data for A) female, and B) male placenta samples.

**Additional file 3.** Principal component analyses. A) female placenta samples; B) male placenta samples; C) male and female samples.

**Additional file 4.** Differentially expressed genes at high versus low U-As adjusted for maternal age at enrollment and batch. A) female placenta, B) male placenta.

**Additional file 5. **qPCR validation of selected differentially expressed genes. RNA was extracted from repeat samples of the same placentas used for the RNA-seq analysis, and qPCR was performed using TaqMan probes designed against a subset of the top 10 differentially expressed genes in female placentas. Error bars show SEM. * *P* < 0.05.

**Additional file 6.** Gene sets enriched with differentially expressed genes at high versus low U-As at FDR < 0.05, in A-B) female and C-D) male fetal placenta, and E) comparison.

**Additional file 7.** Gene set enrichment analysis of arsenic-exposed female fetal placenta. Enrichment map showing gene sets enriched with differentially expressed genes at high versus low arsenic exposure (U-As levels) in female fetal placenta. Clustered gene sets with a significance level of FDR < 0.05 are shown. The “canonical pathways” gene set collection from MSigDB was used. Red circles; upregulated gene sets, blue circles; downregulated gene sets, large yellow circles; gene set clusters, green lines indicate overlapping genes between gene sets; words that appear most frequently in the gene set titles are shown. Singleton gene sets are omitted (see Additional file 6A-B for a complete list of gene sets).

**Additional file 8.** Gene set enrichment analysis of arsenic-exposed male fetal placenta. Enrichment map showing gene sets enriched with differentially expressed genes at high versus low arsenic exposure (U-As levels) in male fetal placenta. Clustered gene sets with a significance level of FDR < 0.05 are shown. The “canonical pathways” gene set collection from MSigDB was used. Red circles; upregulated gene sets, blue circles; downregulated gene sets, large yellow circles; gene set clusters, green lines indicate overlapping genes between gene sets; words that appear most frequently in the gene set titles are shown. Singleton gene sets are omitted (see Additional file 6C-D for a complete list of gene sets).

**Additional file 9.** Gene sets enriched with differentially expressed genes at high versus low birth weight at FDR < 0.05, in A) female and B) male fetal placenta, and C) comparison.

**Additional file 10.** Gene sets enriched in both birth weight and U-As analyses in A) female and B) male fetal placenta at FDR < 0.05. Gene sets showing opposite directions of association in 9A) are asterisked.

## Data Availability

The dataset supporting the conclusions of this article is included within the article and its additional files.

## Abbreviations

EMap: Enrichment map
FDR: False discovery rate
GSEA: Gene set enrichment analysis
NHBCS: New Hampshire Birth Cohort Study
RNA-seq: RNA-sequencing
U-As: Total maternal urinary arsenic concentration (excluding arsenobetaine)
