# Supplementary figures and images for: Transcriptome-wide analysis of changes in the fetal placenta associated with prenatal arsenic exposure in the New Hampshire Birth Cohort Study
Source: Environ Health. 2019 Nov 21;18:100. doi: 10.1186/s12940-019-0535-x (PMC6868717; doi:10.1186/s12940-019-0535-x)

## Slide 1
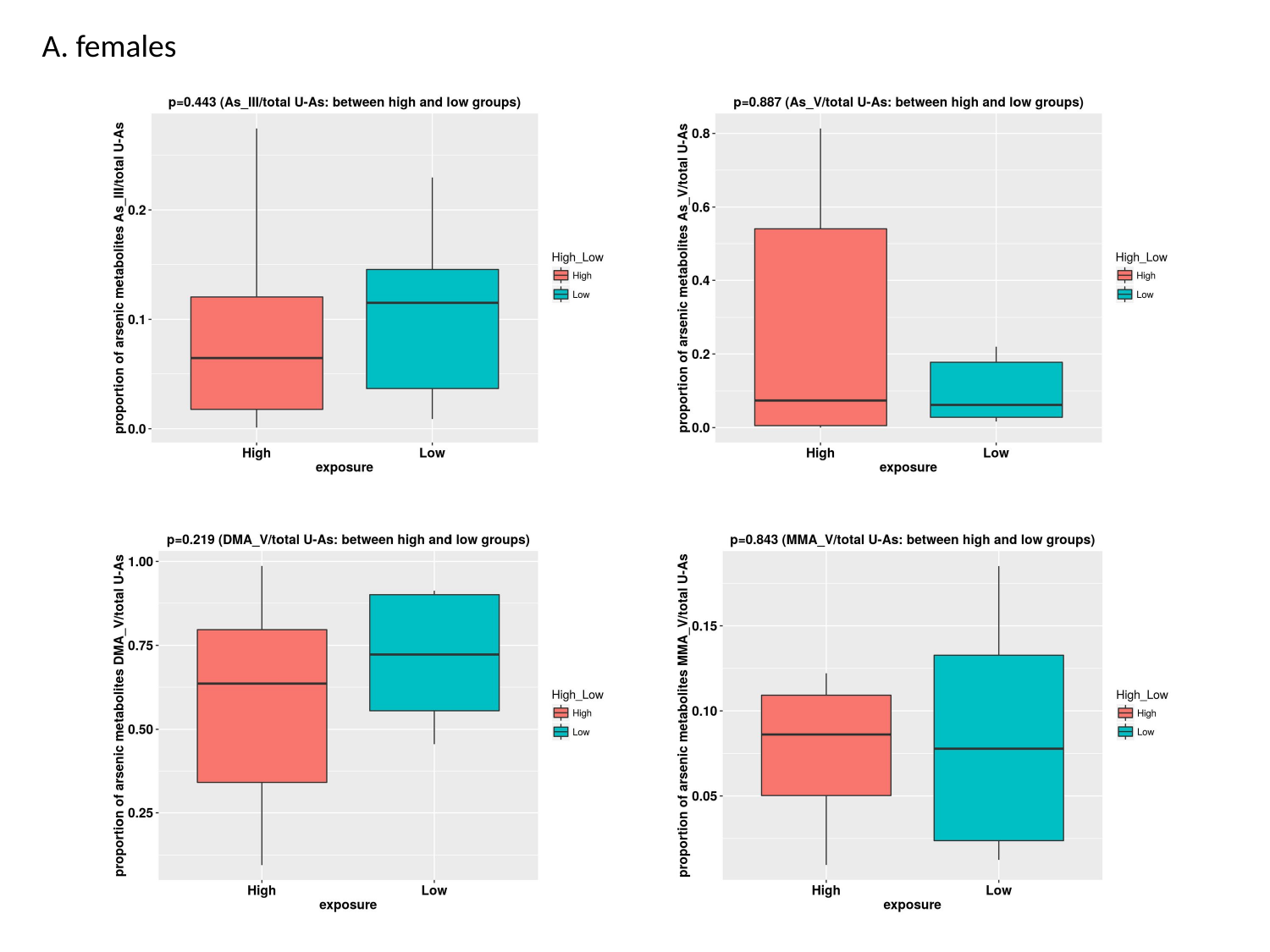

A. females

## Slide 2
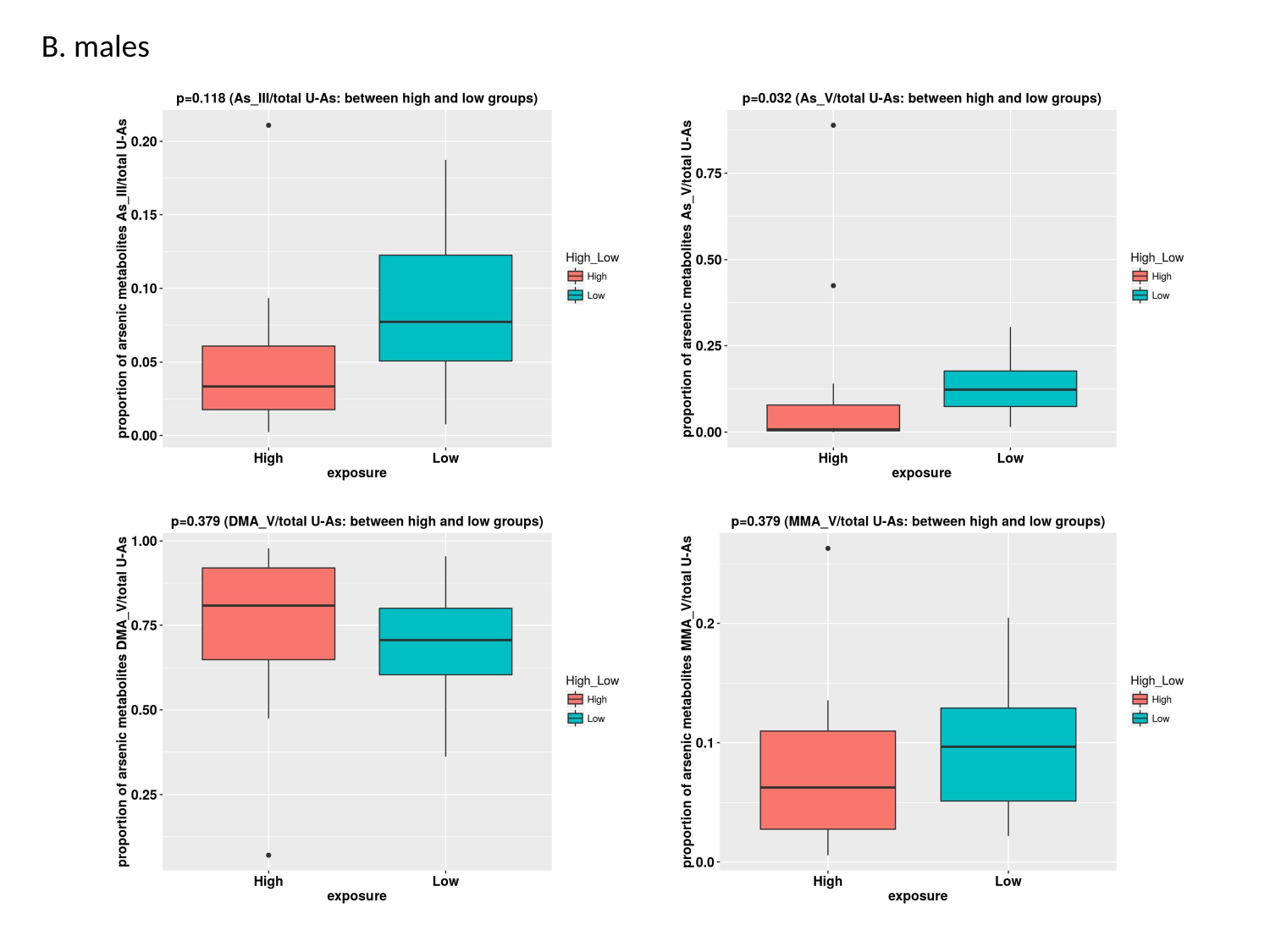

B. males

Supplement: Supplementary file 1 — Additional file 1. Relative proportions of arsenic species in high and low arsenic groups. Upper and lower ends of boxes indicate the 25th and 75th percentiles, respectively, and black band represents the median. Error bars represent minimum and maximum values, excluding outliers, which are depicted as dots. P values are based on a Wilcoxon signed rank test. [file 12940_2019_535_MOESM1_ESM.pptx]

## Slide 1
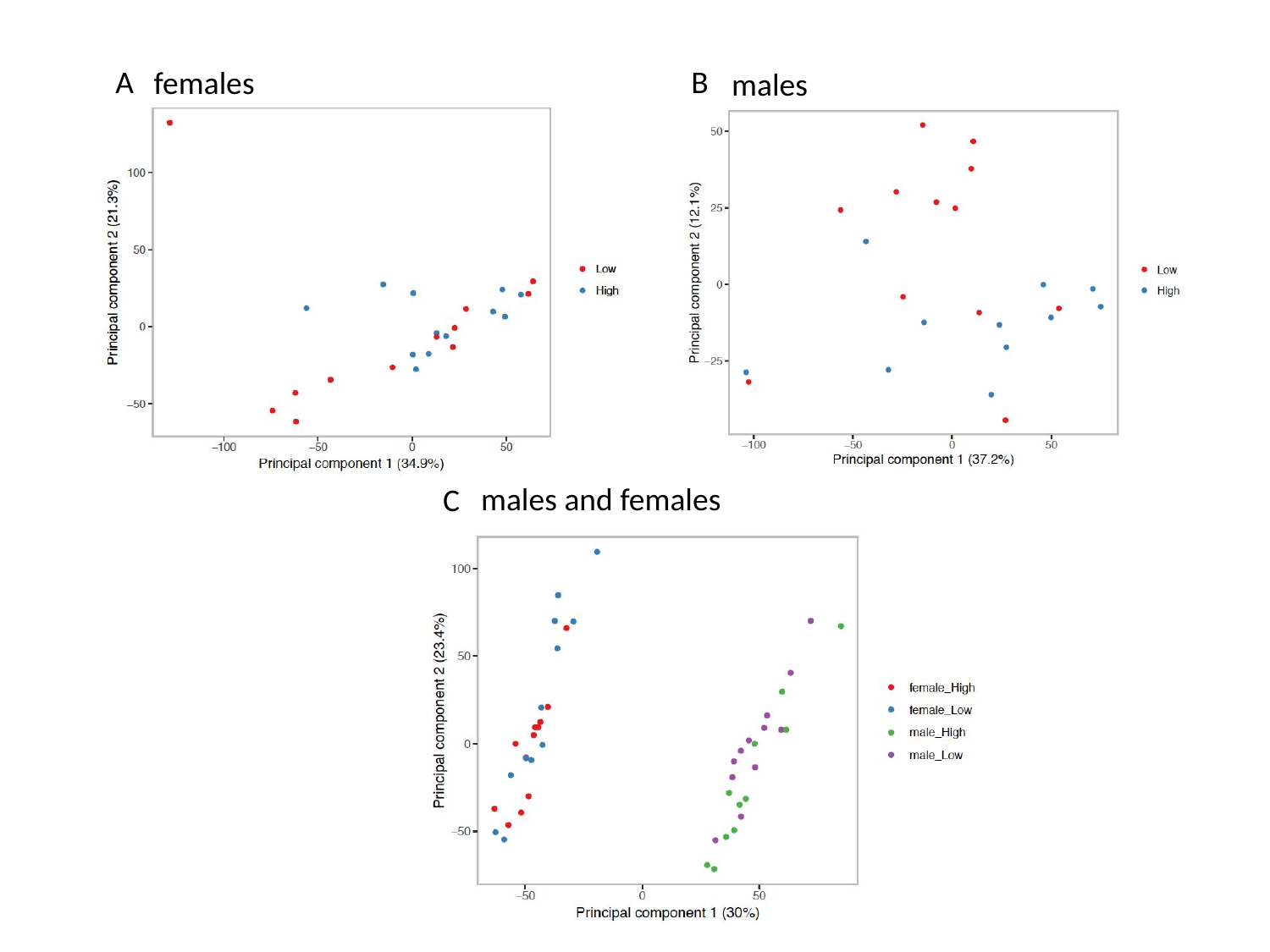

B
A
females
males
males and females
C

Supplement: Supplementary file 3 — Additional file 3. Principal component analyses. A) female placenta samples; B) male placenta samples; C) male and female samples. [file 12940_2019_535_MOESM3_ESM.pptx]

## Slide 1
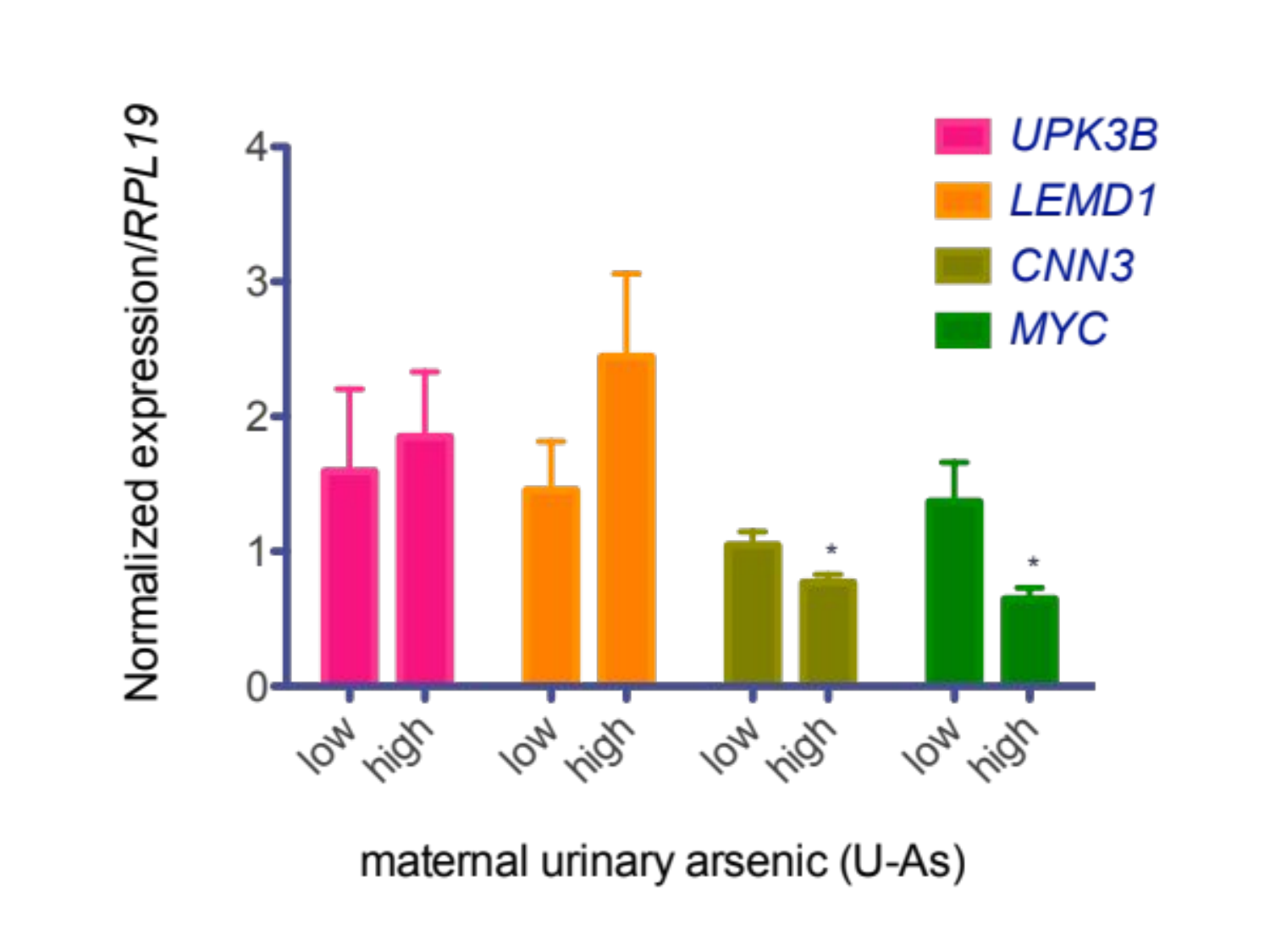

Supplement: Supplementary file 5 — Additional file 5. qPCR validation of selected differentially expressed genes. RNA was extracted from repeat samples of the same placentas used for the RNA-seq analysis, and qPCR was performed using TaqMan probes designed against a subset of the top 10 differentially expressed genes in female placentas. Error bars show SEM. * P < 0.05. [file 12940_2019_535_MOESM5_ESM.pptx]

## Slide 1
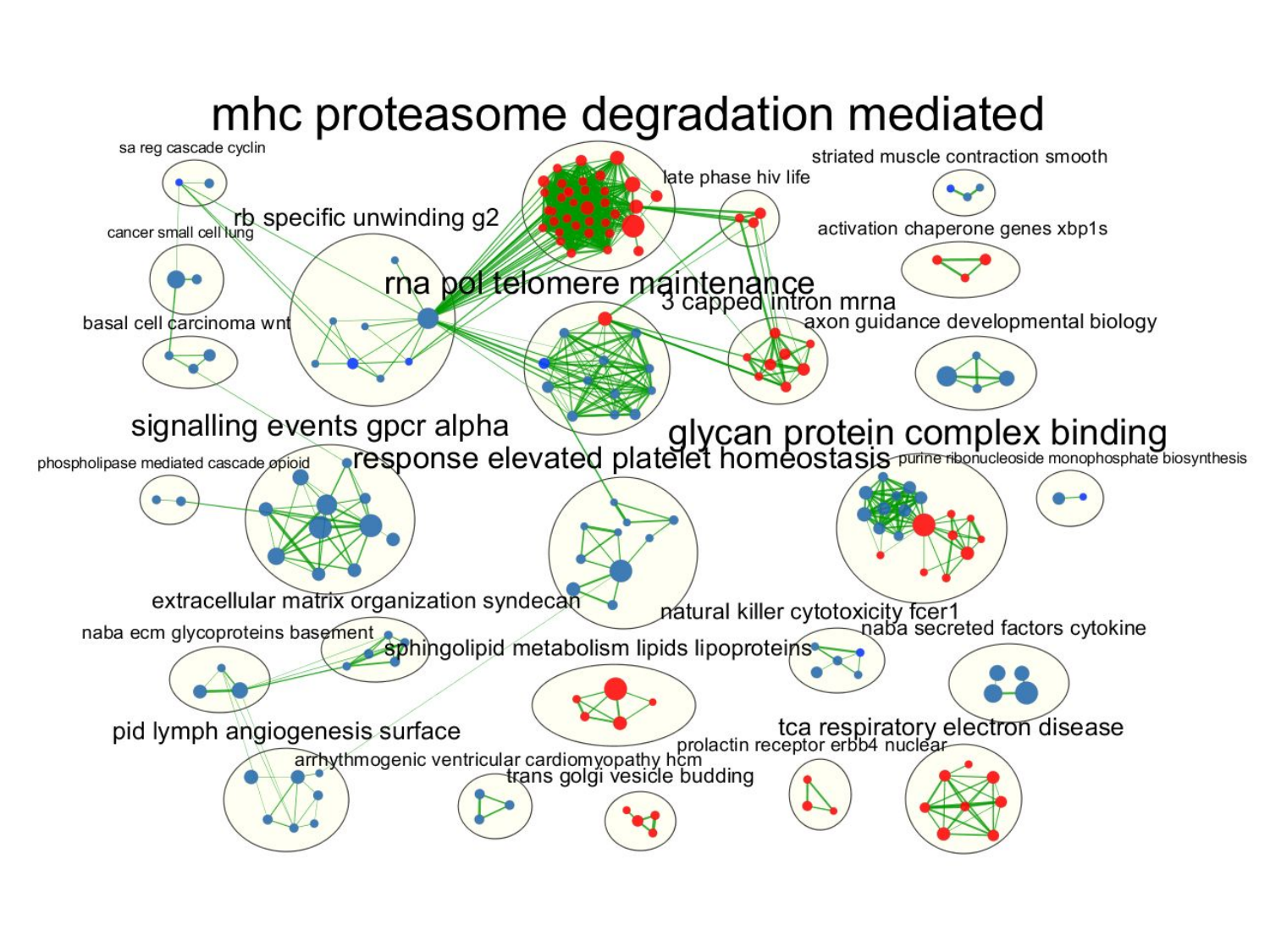

Supplement: Supplementary file 7 — Additional file 7. Gene set enrichment analysis of arsenic-exposed female fetal placenta. Enrichment map showing gene sets enriched with differentially expressed genes at high versus low arsenic exposure (U-As levels) in female fetal placenta. Clustered gene sets with a significance level of FDR < 0.05 are shown. The “canonical pathways” gene set collection from MSigDB was used. Red circles; upregulated gene sets, blue circles; downregulated gene sets, large yellow circles; gene set clusters, green lines indicate overlapping genes between gene sets; words that appear most frequently in the gene set titles are shown. Singleton gene sets are omitted (see Additional file 6A-B for a complete list of gene sets). [file 12940_2019_535_MOESM7_ESM.pptx]

## Slide 1
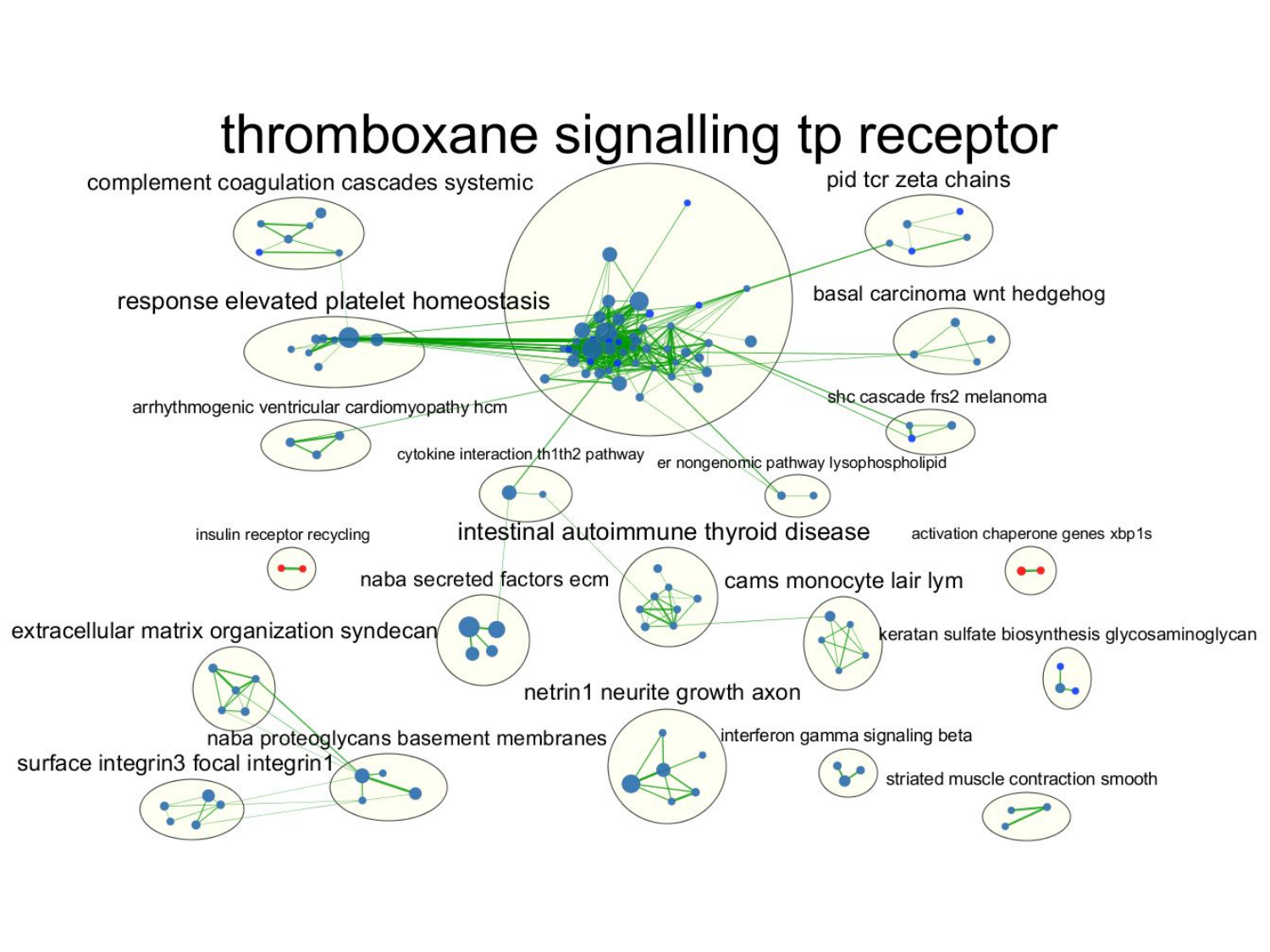

Supplement: Supplementary file 8 — Additional file 8. Gene set enrichment analysis of arsenic-exposed male fetal placenta. Enrichment map showing gene sets enriched with differentially expressed genes at high versus low arsenic exposure (U-As levels) in male fetal placenta. Clustered gene sets with a significance level of FDR < 0.05 are shown. The “canonical pathways” gene set collection from MSigDB was used. Red circles; upregulated gene sets, blue circles; downregulated gene sets, large yellow circles; gene set clusters, green lines indicate overlapping genes between gene sets; words that appear most frequently in the gene set titles are shown. Singleton gene sets are omitted (see Additional file 6C-D for a complete list of gene sets). [file 12940_2019_535_MOESM8_ESM.pptx]
